# Investigation of dynamic responses of skin simulant against fragment impact through experiments and concurrent computational modeling

**DOI:** 10.3389/fbioe.2024.1422685

**Published:** 2024-08-27

**Authors:** Punit Kumar Pandey, S. G. Ganpule

**Affiliations:** ^1^ Department of Mechanical and Industrial Engineering, Indian Institute of Technology Roorkee, Roorkee, India; ^2^ Department of Design, Indian Institute of Technology Roorkee, Roorkee, India

**Keywords:** skin simulant, fragment, impact, threshold velocity, failure, strain rate sensitivity

## Abstract

Perforation of the skin by fragment impact is a key determinant of the severity of an injury and incapacitation during modern asymmetric warfare. Computational models validated against experimental data are thus desired for simulating the responses of a skin simulant against fragment impact. Toward this end, experiments and concurrent computational modeling were used to investigate the dynamic responses of the skin simulant against fragment impact. Fragment simulating projectiles (FSPs) of masses 1.10 g and 2.79 g were considered herein, and the responses of the skin simulant were investigated in terms of the threshold velocity, energy density, peak displacement, and failure mechanisms. The results illustrate numerous salient aspects. The skin simulant failure involved cavity shearing followed by elastic hole enlargement, and these results were sensitive to the strain rate. The best agreement between the simulated and experimental results was achieved when the input stress–strain curves to the simulation were based on the full spectrum of strain rates. When a single stress–strain curve corresponding to a specific strain rate was used as the input, the threshold velocity and peak displacement of the skin simulant were either underpredicted or overpredicted depending on the strain rate considered. The threshold velocity was also sensitive to the input failure strain; here, the best agreement was obtained when the failure strain was based on the theoretical limiting strain. When the FSP materials were changed to plastics, the threshold velocities increased by up to 33%; however, the energy densities and generated stresses exceeded the contusion and laceration thresholds of the skin.

## 1 Introduction

The skin is the outermost part of the human body and acts as an initial barrier against any external loading (Limbert, 2017; Chanda, 2018; Wahlsten et al., 2019). Perforation of the skin by high-velocity projectiles, such as bullets and fragments, is considered sufficient for human incapacitation on the battlefield (Allen and Sperrazza, 1956; Henderson, 2010; Breeze and Clasper, 2013; Zecevic et al., 2015). The majority of skin-penetrating combat injuries are caused by fragments generated from ammunition such as improvised explosive devices, grenades, antipersonnel warheads, and explosive mines (Bowyer et al., 1995; Champion et al., 2003; Breeze and Clasper, 2013; Carr et al., 2017; Regasa et al., 2018). Thus, skin or skin simulant response against fragment impact is a topic of considerable interest.

The ballistic responses of skin or skin simulants against fragment impact are typically evaluated experimentally by launching fragments on skin or skin simulants at high velocities (Sperrazza and Kokinakis, 1968; Breeze and Clasper, 2013; Breeze et al., 2013; Hazell, 2022). Conducting large numbers of such ballistic experiments is challenging and costly. Thus, robust computational models benchmarked against experiments are desired as alternatives (Breeze and Clasper, 2013; Breeze et al., 2014). Accordingly, existing constitutive models of soft materials can be calibrated using the ballistic experiment data; these calibrated constitutive models and their material parameters or stress–strain response curves may be used in higher-order computational models, such as 3D head and 3D anatomically accurate computational models. Higher-order models have greater utility in simulating real-life scenarios, such as penetrating ballistic impact.

There are several challenges in simulating the responses of skin or skin simulants under high loading rates. First, the available experimental stress–strain responses of the skin or skin simulants at high strain rates (>10^1^ s^-1^) are sparse. Most of the available data are acquired at quasi-static strain rates (Joodaki and Panzer, 2018; Jor et al., 2013; Kalra et al., 2016; Sachs et al., 2021). A few investigations provide stress–strain response at high strain rates under compression (Shergold et al., 2006; Joodaki and Panzer, 2018); such data are not readily available for skin under tension, and only one study (Khodadadi et al., 2019b) provides the simulant data under tension (albeit not up to failure).

Obtaining data under skin tension is more challenging than compression owing to several technical challenges in the tensile testing of such soft materials on the split Hopkinson pressure bar (SHPB). The specimen geometries, such as dog-bone shape, and critical connections between the specimen and input/output bars pose significant technical difficulties during tensile loading (Chen, 2016; Guo and Wang, 2020; Upadhyay et al., 2021). Designing and attaching grips to the tensile specimens to effectively transfer uniaxial loads to their gage sections (i.e., measurement zones) are particularly tricky. Achieving appropriate load transfer without inducing any damage to the specimen edges and minimizing the edge effects thus becomes critical. Improper gripping techniques can also lead to the development of a triaxial stress state within the specimen (Upadhyay et al., 2021). Moreover, fully characterizing the tensile properties of soft materials (up to the failure point) often requires large tensile deformations, which are challenging to achieve within the limitations of the SHPB system (Siviour and Jordan, 2016).

First, high-strain-rate experimental data of skin or skin simulants required for constitutive modeling are lacking, and most of the computational modeling of skin or skin simulant responses in literature use either quasi-static data or data obtained at a single strain rate (see Joodaki and Panzer (2018) and the references therein). Second, the experimental data for relevant loading scenarios (e.g., fragment or bullet impact) desired for model calibration and validation are scant. Toward this end, to bridge the aforementioned gaps, we simulated the responses of a skin simulant under fragment impact. The high-strain-rate stress–strain responses of the skin simulant under tension were retrieved from literature, and the data were extrapolated adequately up to the failure strains (estimated theoretically). The sensitivity of the model to the material parameters (i.e., input stress–strain curves and failure strain), thickness of the skin simulant, as well as shape, size, and material of the fragment were investigated.

## 2 Method

### 2.1 Experiments

**FIGURE 1:**
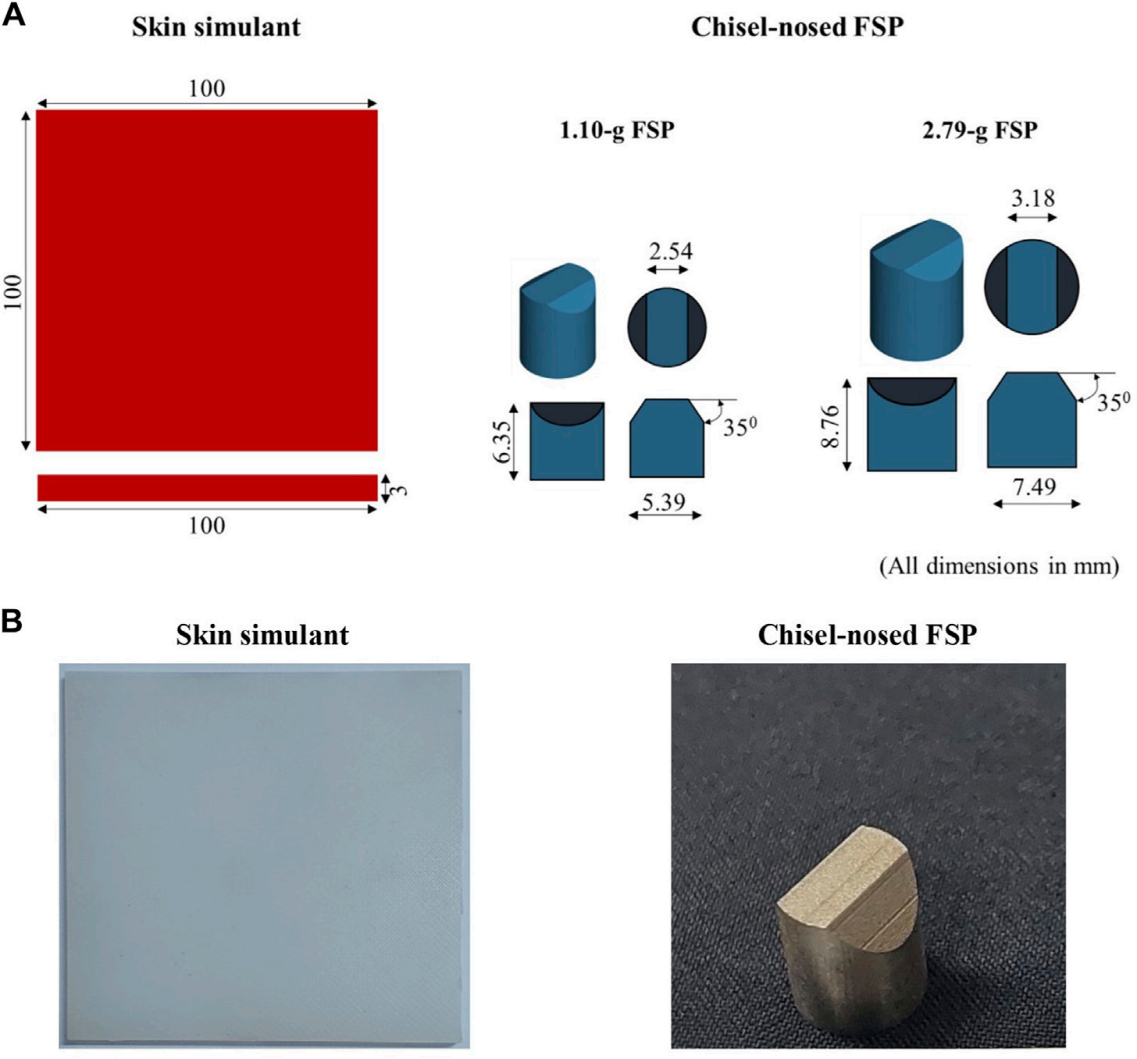
Skin simulant and chisel-nosed FSPs: **(A)** schematics depicting dimensions and **(B)** actual photographs.

Experiments were conducted to investigate the responses of the skin simulant to fragment impact. These data were used to validate the numerical model. A two-part silicone substance (Smooth-On, Inc., Macungie, PA) with a shore hardness of 30A was used as the skin simulant; this material exhibits a stress–strain response similar to human skin (Chanda et al., 2017; Chanda and Upchurch, 2018; Marechal et al., 2021). The skin simulant was prepared in the form of a rectangular plate of size 100 mm × 100 mm × 3 mm, where the 3 mm thickness was selected based on the average thickness documented for human skin in literature (Sperrazza and Kokinakis, 1968; Yoganandan and Pintar, 1997; Chanda, 2018). Mild-steel chisel-nosed fragment simulating projectiles (FSPs) of masses 1.10 g and 2.79 g (Figure 1) were manufactured according to the standard sizes specified in NATO STANAG 2920 (NATO, 2003; Bolduc and Jager, 2016).

**FIGURE 2:**
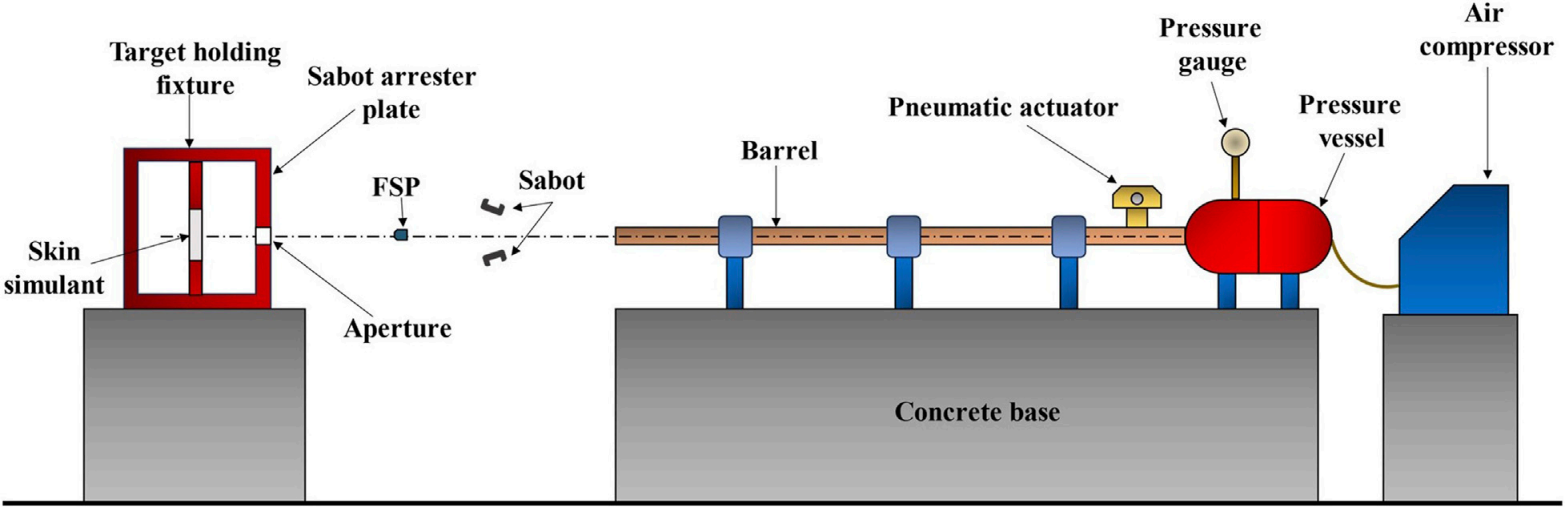
Front-view schematic illustration of the pneumatic gas gun setup.

A pneumatic gas gun setup (Figure 2) was used to conduct the experiments. The setup comprised an air compressor, a pressure vessel, a pressure gage, an electric actuator, and a seamless barrel. To accommodate FSPs of different sizes in a fixed-diameter barrel, a split sabot having an outer diameter equal to the diameter of the barrel with a cavity tailored to the specific FSP size was used. The sabots were accelerated by the sudden release of compressed air from the pressure vessel. After exiting the barrel, the sabot opened due to air drag, and the FSP moved faster than the sabot owing to its lower mass and lower air drag. Upon traveling further, the sabot was arrested by the sabot arrester plate, allowing the FSP to pass through an aperture and impact the skin simulant.

The required velocities of the FSPs were attained by adjusting the effective barrel length (i.e., distance between the sabot and open end of the barrel) and compressed air pressure in the pressure vessel. The FSPs were launched onto the skin simulant in the velocity range of 49−170 m s^–1^, with a variation of ±5 m s^–1^ for a given effective barrel length and air pressure. A high-speed camera (Phantom v411, Vision Research, Inc., Wayne, NJ) was installed to capture the ballistic test events; the captured high-speed images were used to obtain the velocities of the FSPs. The frame rate of the high-speed camera was 16,000 frames per second. A total of 46 impact experiments were performed. Table 1 presents the impact velocities (V_i_) and corresponding results of the impact events (i.e., perforation or non-perforation) for the two FSPs. Each sample was impacted once to avoid the response effects from previous loading.

**TABLE 1:** Observed results (perforation or non-perforation) at various impact velocities (V_i_) for the two FSPs.

1.10 g FSP			2.79 g FSP		
Exp. No.	V_i_ (m/s)	Result	Exp. No.	V_i_ (m/s)	Result
1	41	Non-perforation	1	49	Non-perforation
2	53	Non-perforation	2	56	Non-perforation
3	62	Non-perforation	3	57	Non-perforation
4	67	Non-perforation	4	59	Non-perforation
5	68	Non-perforation	5	63	Non-perforation
6	71	Non-perforation	6	65	Non-perforation
7	71	Non-perforation	7	66	Non-perforation
8	76	Non-perforation	8	68	Perforation
9	79	Non-perforation	9	69	Perforation
10	83	Non-perforation	10	76	Perforation
11	89	Non-perforation	11	76	Perforation
12	88	Perforation	12	81	Perforation
13	88	Perforation	13	85	Perforation
14	88	Perforation	14	86	Perforation
15	90	Perforation	15	89	Perforation
16	90	Perforation	16	93	Perforation
17	92	Perforation	17	93	Perforation
18	92	Perforation	18	94	Perforation
19	95	Perforation	19	97	Perforation
20	95	Perforation	20	97	Perforation
21	96	Perforation	21	100	Perforation
22	99	Perforation	22	108	Perforation
23	136	Perforation	23	135	Perforation

### 2.2 Finite-element model

#### 2.2.1 Finite-element discretization

**FIGURE 3:**
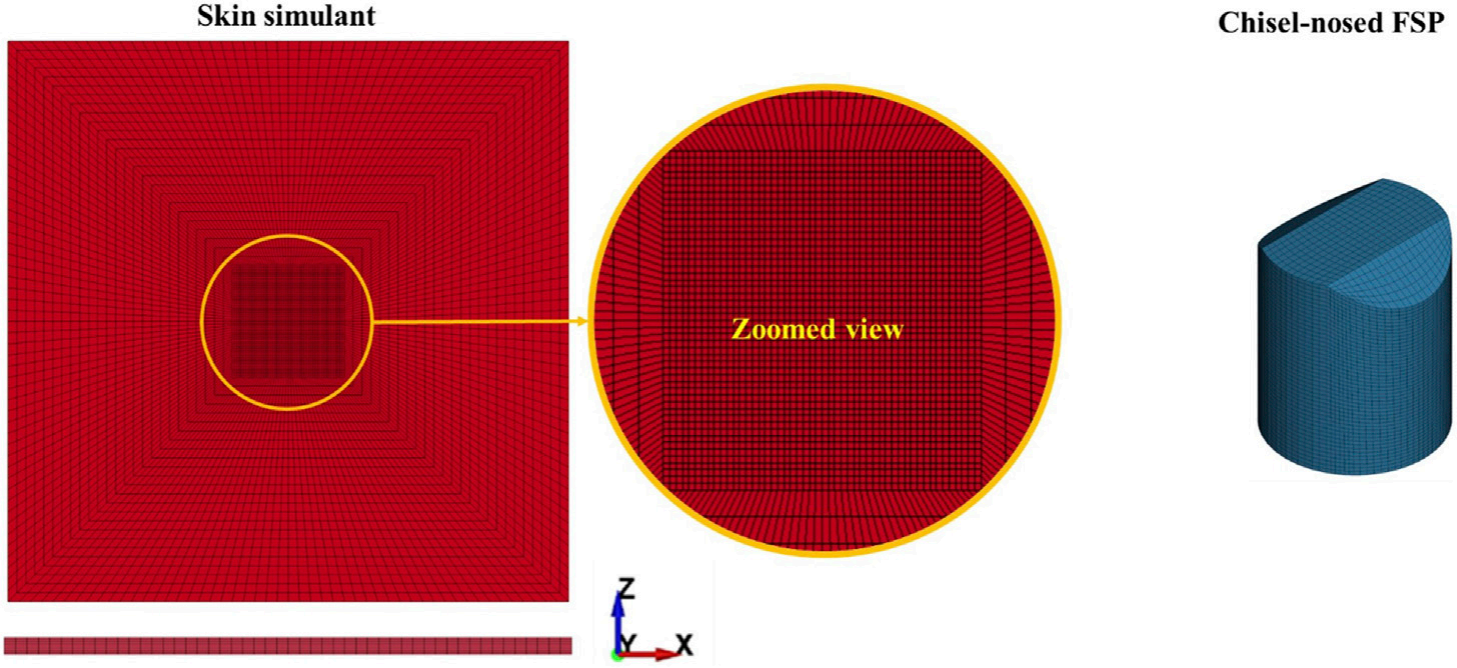
Finite-element discretization of the skin simulant and FSP.

A finite-element model was considered to simulate the responses of the skin simulant against fragment impact. The model was built to mimic the experiments described in Section 2.1. The skin simulant and FSPs were discretized using linear hexahedral elements with reduced integration (ELFORM 1 of LS-DYNA) (Figure 3). The central part (i.e., impact zone) of the skin simulant having dimensions of 10 mm × 10 mm × 3 mm was finely meshed with elements of size 0.4 mm × 0.4 mm × 0.4 mm. The mesh converged (<5% difference in the residual velocity) at this mesh resolution (Supplementary Figure S1). To optimize the computational efficiency, the mesh size was increased gradually toward the outer boundaries of the skin simulant plate up to a mesh size of 1.6 mm × 2.0 mm × 0.4 mm. This resulted in 60,000 elements for the skin simulant. All four edges of the skin simulant were fully constrained to replicate the experimental boundary conditions. The 1.10 g and 2.79 g FSPs were meshed with elements of size 0.25 mm × 0.25 mm × 0.25 mm, resulting in 24,288 and 50,880 hexahedral elements, respectively.

A two-way, surface-to-surface eroding contact was used to model the interactions between the skin simulant and FSPs. The eroding contact is useful when there is a probability of element deletion upon meeting the failure criterion. The contact surfaces in the eroding contact were continuously updated to account for the element deletions (LSTC, 2021). Simulations were performed using a non-linear, transient, explicit dynamic scheme in which the initial velocity of the FSPs was set according to the experimental impact velocity (V_i_). The simulations were then performed with 32 processors (Intel^®^ Xeon^®^ Gold 6,134, processor speed 3.00 GHz) and a massively parallel processing (MPP) solver in LS-DYNA V971 R4.7 (LSTC, 2021). Time steps of the order of 10^−9^ s were used in the simulations to ensure stability; each simulation iteration required ∼22 min of CPU time for a total simulation time of 1.5 ms.

#### 2.2.2 Constitutive model of the skin simulant

The skin simulant was modeled using a phenomenological material; this material model is based on the Ogden hyperelasticity (Ogden, 1972), whose energy function is given by

$$W=\sum_{i=1}^{3}\sum_{j=1}^{m}\frac{\mu_{j}}{\alpha_{j}}\;\left(\;\lambda_{i}^{{*\alpha }_{j}}-1\right)+K\;\left(\;J-1-\ln J\right),$$ (1)

where μ (shear modulus), α (strain hardening exponent), and K (bulk modulus) are the material constants; λ is the principal stretch, $\lambda^{*}=\lambda J^{-1/3}$ is the deviatoric principal stretch, and $J=\lambda_{1}\lambda_{2}\lambda_{3}$ is the Jacobian.

The principal stresses can be computed as

$$\sigma_{i}=\frac{1}{\lambda_{p}\lambda_{q}}\;\frac{\partial W}{\partial \lambda_{i}.}$$ (2)

The subscripts p and q in Equation 2 refer to the two coordinate directions perpendicular to i. Substituting Equation 1 into Equation 2, we have

$$\sigma_{i}=\frac{1}{\lambda_{p}\lambda_{q}}\;\frac{\partial W}{\partial \lambda_{i}}=\sum_{j=1}^{m}\frac{\mu_{j}}{J}\;\left[\;\lambda_{i}^{{*\alpha }_{j}}-\sum_{p=1}^{3}\frac{\lambda_{p}^{{*\alpha }_{j}}}{3}\right]+K\;\frac{J-1}{J}.$$ (3)

Kolling et al. (2007) proposed the equivalent forms of Equations 1, 3 as follows

$$W=\sum_{i=1}^{3}g\;\left(\lambda_{i}\right)+K\;\left(\;J-1-\ln J\right).$$ (4)

$$\sigma_{i}=\frac{1}{J}\;\left(f\left(\lambda_{i}\right)-\frac{1}{3}\sum_{j=1}^{3}f\left(\lambda_{j}\right)\right)+K\;\frac{J-1}{J}.$$ (5)

Equations 4, 5 facilitate the calculation of $g\left(\lambda \right),$ $f\left(\lambda \right),$ W, and σ directly from the tabulated data without the need to explicitly calculate the fitting parameters μ and α. This approach is especially convenient when modeling rate-dependent hyperelastic behaviors (Kolling et al., 2007). Next, $g\left(\lambda_{i}\right)\text{ and}$ $f\left(\lambda_{i}\right)$ can be estimated from the uniaxial stress–strain data (Equations 6–14). $g\left(\lambda_{i}\right)$ is defined as

$$g\left(\lambda_{i}\right)=\sum_{j=1}^{m}\frac{\mu_{j}}{\alpha_{j}}\;\left(\lambda_{i}^{{*\alpha }_{j}}-1\right).$$ (6)

$g\left(\lambda \right)$ can be written in terms of $W_{u}\left(\lambda \right)$ , where $W_{u}\left(\lambda \right)$ is the deformation energy per unit undeformed volume expressed in terms of the uniaxial engineering stress ( $\left.\sigma_{u}\right)$ and uniaxial engineering strain ( $\left.\varepsilon_{u}\right)$ as follows:

$$W_{u}\left(\lambda \right)=\int_{0}^{\varepsilon }\sigma_{u}d\varepsilon_{u}=\int_{0}^{\lambda }\sigma_{u}d\lambda .$$ (7)

Equation 1 can be evaluated for the uniaxial test. For a nearly incompressible material, J≈1 and $\lambda_{p}^{*}\approx \lambda_{q}^{*}\approx {\lambda_{i}^{*}}^{-\frac{1}{2}}$ . Substituting these in Equation 1, we have

$$W_{u}\left(\lambda_{i}\right)=\sum_{j=1}^{m}\frac{\mu_{j}}{\alpha_{j}}\;\left(\;\lambda_{i}^{{*\alpha }_{j}}-1\right)+2\sum_{j=1}^{m}\frac{\mu_{j}}{\alpha_{j}}\left(\lambda_{i}^{*-\frac{\alpha_{j}}{2}}-1\right),$$ (8)

$$W_{u}\left({\lambda_{i}}^{-\frac{1}{2}}\right)=\sum_{j=1}^{m}\frac{\mu_{j}}{\alpha_{j}}\;\left(\;\lambda_{i}^{*-\frac{\alpha_{j}}{2}}-1\right)+2\sum_{j=1}^{m}\frac{\mu_{j}}{\alpha_{j}}\left(\lambda_{i}^{*\frac{\alpha_{j}}{4}}-1\right),$$ (9)

and hence

$$g\left(\lambda_{i}\right)=W_{u}\left(\lambda_{i}\right)-2W_{u}\left({\lambda_{i}}^{-\frac{1}{2}}\right)+4W_{u}\left({\lambda_{i}}^{\frac{1}{4}}\right)-\ldots$$ (10)

Equation 10 represents an infinite series. However, the terms of the series can be truncated upon meeting a desired tolerance. For

$$\left|\lambda_{i}^{{\left(-1/2\right)}^{x}}-1\right|\leq 0.01$$

$$g\left(\lambda_{i}\right)=W_{u}\left(\lambda_{i}-1\right)+\sum_{x=1}^{\infty }{\left(-2\right)}^{x}\;W_{u}\left(\lambda_{i}^{{\left(-1/2\right)}^{x}}-1\right).$$ (11)

Next, $f\left(\lambda_{i}\right)$ is evaluated as

$$f\left(\lambda_{i}\right)=\sum_{j=1}^{m}\mu_{j}\lambda_{i}^{{*\alpha }_{j}.}$$ (12)

$f\left(\lambda_{i}\right)$ can be written in terms of $\sigma_{u}$ as (for the detailed derivation, please refer to Kolling et al. (2007))

$$f\left(\lambda_{i}\right)=\lambda_{i}\sigma_{ui}\left(\lambda_{i}-1\right)+\lambda_{i}^{\left(-\frac{1}{2}\right)}\sigma_{ui}\left(\lambda_{i}^{\left(-\frac{1}{2}\right)}-1\right)+\lambda_{i}^{\left(\frac{1}{4}\right)}\sigma_{ui}\left(\lambda_{i}^{\left(\frac{1}{4}\right)}-1\right)+\ldots$$ (13)

where the terms of the series in Equation 13 can be truncated upon meeting a desired tolerance. For

$$\left|\lambda_{i}^{{\left(-1/2\right)}^{x}}-1\right|\leq 0.01$$

$$f\left(\lambda_{i}\right)=\lambda_{i}\sigma_{ui}\left(\lambda_{i}-1\right)+\sum_{x=1}^{\infty }\lambda_{i}^{{\left(-1/2\right)}^{x}}\sigma_{ui}\times \left(\lambda_{i}^{{\left(-1/2\right)}^{x}}-1\right).$$ (14)

Equation 5 provides an exact fit to the experimental uniaxial stress–strain curves. Kolling et al. (2007) showed that although this model needs only the uniaxial stress–strain response, it yields satisfactory results for various types of loading and is not limited to only uniaxial loading.

**FIGURE 4:**
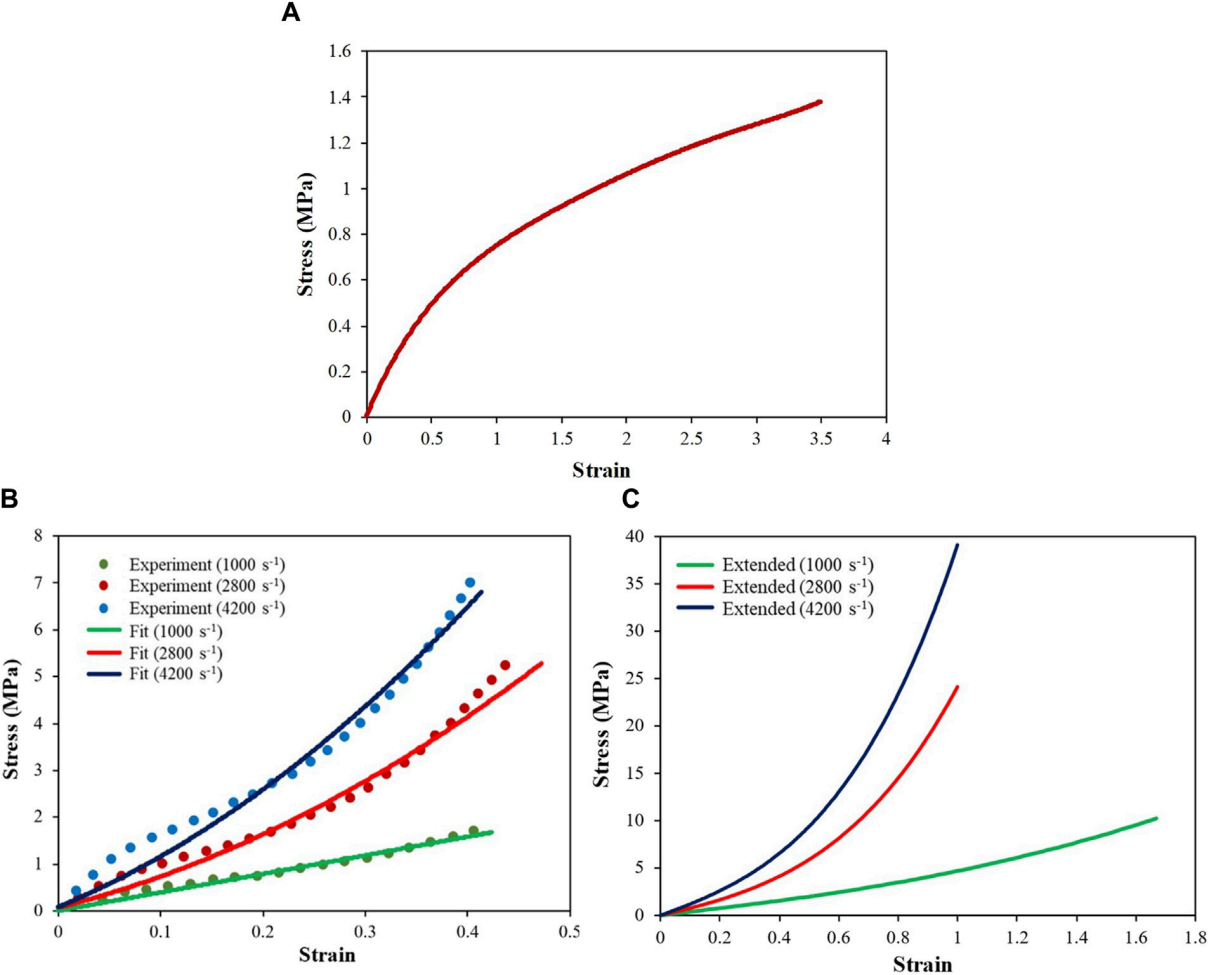
Engineering stress–strain responses of the skin simulant at different strain rates: **(A)** quasi-static response; **(B)** high-strain-rate responses fitted with stress–strain curves using experimental data (Khodadadi et al., 2019b); **(C)** extended stress–strain curves up to failure strain.

Equation 5 considers the strain rate effect by permitting the users to input several uniaxial engineering stress–strain curves (in tabulated form), each corresponding to a different strain rate. When the strain rate in the simulation differs from the input strain rate (and associated stress–strain response), the model determines the constitutive behavior by interpolating between the input stress–strain curves. The model described above was implemented as MAT-181 in LS-DYNA (LSTC, 2021). The rate-dependent stress–strain response under uniaxial tension was adopted from literature (Khodadadi et al., 2019a; Khodadadi et al., 2019b), and a similar response was assumed for compression. At the quasi-static strain rate (Figure 4A), the experimental stress response was available up to failure strain. However, at higher strain rates (i.e., 1,000 s^-1^, 2,800 s^-1^, and 4,200 s^-1^), the experimental stress response (Figure 4B) was available up to a strain of ∼0.40. We extended each stress–strain curve (Figure 4C) corresponding to the higher strain rates up to the limiting failure strain by fitting the experimental data using Equation 3. The limiting or maximum possible failure strain at each strain rate was estimated by fitting the experimental data using a Gent model (details below). These extended stress–strain data were used as the inputs to the simulation. The stress–strain data at each strain rate were input in tabular form. The strain energy and principal stresses at each time step were calculated using Equations 4, 5.

Since the experimental data up to failure strain were not available at high strain rates, we estimated the limiting strains by fitting the experimental stress–strain curve at each strain rate using the Gent (Gent, 1996; Rashid et al., 2014) hyperelastic strain energy function (Equation 15). Note that we used the same experimental stress–strain curves at each strain rate for both the Ogden and Gent models. The Gent model facilitates estimation of the limiting chain extensibility (Horgan and Saccomandi, 2003; Upadhyay et al., 2021):

$$W=-\frac{\mu }{2}J_{m}\ln\;\left(1-\frac{I_{1}-3}{J_{m}}\right),$$ (15)

where the shear modulus μ and chain extensibility parameter $J_{m}$ are the fitting parameters; $J_{m}$ denotes the maximum value of $I_{1}-3$ , which represents the fully stretched state (i.e., limiting state, W →∞ ).

$$∴\;J_{m}={\left(I_{1}\right)}_{m}-3.$$ (16)

For the uniaxial case,

$${\;\left(I_{1}\right)}_{m}=\lambda_{m}^{2}+\frac{2}{\lambda_{m},}$$ (17)

$$\lambda_{m}=1+\varepsilon_{m,}$$ (18)

where $\lambda_{m}$ is the maximum or limiting stretch and $\varepsilon_{m}$ is the limiting strain. Once $J_{m}$ is determined, $I_{1m}$ , $\lambda_{m}$ , and $\varepsilon_{m}$ can be obtained using Equations 16–18. Since $\varepsilon_{m}$ represents the strain at the fully stretched state of the material, we refer to $\varepsilon_{m}$ as the failure strain. The estimated failure strain at each strain rate is presented in Table 2.

**TABLE 2:** Estimated failure strains from the Gent model.

Strain rate (s^-1^)	Failure strain, $\varepsilon_{m}$ (mm mm^–1^)
1,000	1.67
2,800	1.02
4,200	1

In addition to the aforementioned stress–strain data, density and bulk modulus values of 1,080 kg m^–3^ and 2.5 GPa, respectively, were used (Muhr, 2005; Smooth-on). The FSPs were modeled as linear and elastic components, and mild steel was used as the FSP material unless stated otherwise.

### 2.3 Ballistic response estimation

The ballistic responses of the skin simulant were estimated through both experiments and simulations by evaluation the key metrics, namely, threshold velocity (V_th_), threshold energy (E_th_), energy density (E_th_/A), residual velocity (V_r_), and peak deformation of the skin simulant.

#### 2.3.1 Threshold velocity (V_th_)

V_th_ is defined as the minimum FSP velocity required to induce perforation. In the simulation, V_th_ was obtained through an iterative process, where the FSP velocity was incremented by 1 m s^–1^ until perforation was observed. In the experiments, the FSPs were launched with a range of impact velocities, resulting in both perforation and non-perforation of the skin simulant. V_th_ was subsequently determined based on these experimental conditions using a statistical approach in accordance with the NATO STANAG 2920 standard (NATO, 2003). V_th_ was calculated from the arithmetic mean of six impact velocities to account for the experimental scatter. These six velocities comprised three minimum velocities that caused perforation and three maximum velocities that did not cause perforation.

#### 2.3.2 Threshold energy (E_th_) and energy density (E_th_/A)

Here, E_th_ is the kinetic energy corresponding to V_th_ of the respective FSP. Then, E_th_/A is the ratio of E_th_ to the cross-sectional area of the FSP (A). Thus, E_th_/A normalizes the threshold energy of the FSP by its cross-sectional area. The energy density is a particularly useful metric for comparing the threshold energies across multiple projectiles.

#### 2.3.3 Residual velocity (V_r_)

V_r_ is the velocity of the FSP after complete perforation of the skin simulant. A comparative analysis of V_r_ was conducted across a range of V_i_ values in both the experiments and simulations.

#### 2.3.4 Peak deformation of the skin simulant

The peak deformation of the skin simulant was quantified by the maximum deformation until the onset of failure for the perforation cases and until unloading for the non-perforation cases. To visually represent the peak deformation in the experiment images, the stretched part of the skin simulant was highlighted with red shading. This technique was employed for better visualization due to blurring of the high-speed images after magnification. A detailed description of the shading protocol is provided in Supplementary Figure S2.

### 2.4 Parametric studies

The sensitivity of the strain rate to the response of the skin simulant was studied using a full spectrum (i.e., quasi-static, 1,000 s^-1^, 2,800 s^-1^, and 4,200 s^-1^) of stress–strain curves and a single stress–strain curve (corresponding to the specific strain rate) as the inputs. To investigate the effects of the FSP material, two other types of FSPs made of ball bearing (BB) and polyvinyl chloride (PVC) plastics were considered. These FSPs are used as less lethal projectiles during law enforcement operations (Pavier et al., 2015; Jin et al., 2019). The material properties of the various FSPs are tabulated in Table 3. Furthermore, to investigate the influence of skin thickness, we varied the thickness of the simulant in the range of 1–5 mm (Laurent et al., 2007; Joodaki and Panzer, 2018; Chen et al., 2020; Fenton et al., 2020) in increments of 1 mm. For these parametric studies, the FSP of mass 1.10 g was used.

**TABLE 3:** Material properties of the FSPs.

Material	Density (kg m^–3^)	Elastic modulus (MPa)	Poisson’s ratio
Mild steel^a^ (Deka et al., 2008)	7,860	210,000	0.28
BB plastic (Jin et al., 2019)	2,010	2,320	0.30
PVC plastic (Pavier et al., 2015)	1,340	2,300	0.30

^a^ For all simulations (except parametric studies), the FSP material was mild steel.

## 3 Results

### 3.1 Ballistic responses of the skin simulant

The ballistic responses of the skin simulant were investigated in terms of the V_th_, E_th_/A, V_r_, deformation, and failure pattern. For each of the aforementioned parameters, the experimental and numerical results are depicted and compared.

#### 3.1.1 Threshold velocity (V_th_), energy density (E_th_/A), and residual velocity (V_r_)

**FIGURE 5:**
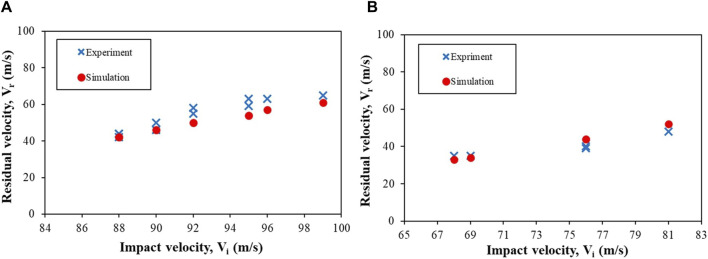
Plots depicting the impact velocity (V_i_) vs. residual velocity (V_r_) of the FSPs having masses **(A)** 1.10 g and **(B)** 2.79 g.

Table 4 shows the V_th_ and E_th_/A values for the 1.10 g and 2.79 g FSPs. The V_th_ and E_th_/A decreased by ∼29% and ∼33%, respectively, as the mass of the FSP increased from 1.10 g to 2.79 g. The differences in V_th_ and E_th_/A between the experiments and simulations were within ∼10%. Interestingly, in absolute terms, the E_th_/A value was within a narrow range of 0.12–0.18 J mm^–2^. A reasonable agreement (within ∼15%) between the experimental and simulated values was obtained for V_r_ as well (Figure 5). Note that at all velocities below V_th_, the FSPs did not perforate the target, resulting in V_r_ = 0. Therefore, only velocities that caused perforation of the skin simulant are included in Figure 5.

**TABLE 4:** Threshold velocities (V_th_) and energy densities (E_th_/A) of the skin simulant for the 1.10 g and 2.79 g FSPs.

FSP		V_th_ (m/s)		E_th_/A (J mm^2^)	
Mass (g)	Cross-sectional area, A (mm^2^)	Experiment	Simulation	Experiment	Simulation
1.10	22.78	86	86	0.18	0.18
2.79	44.11	68	61	0.15	0.12

#### 3.1.2 Deformation of the skin simulant

**FIGURE 6:**
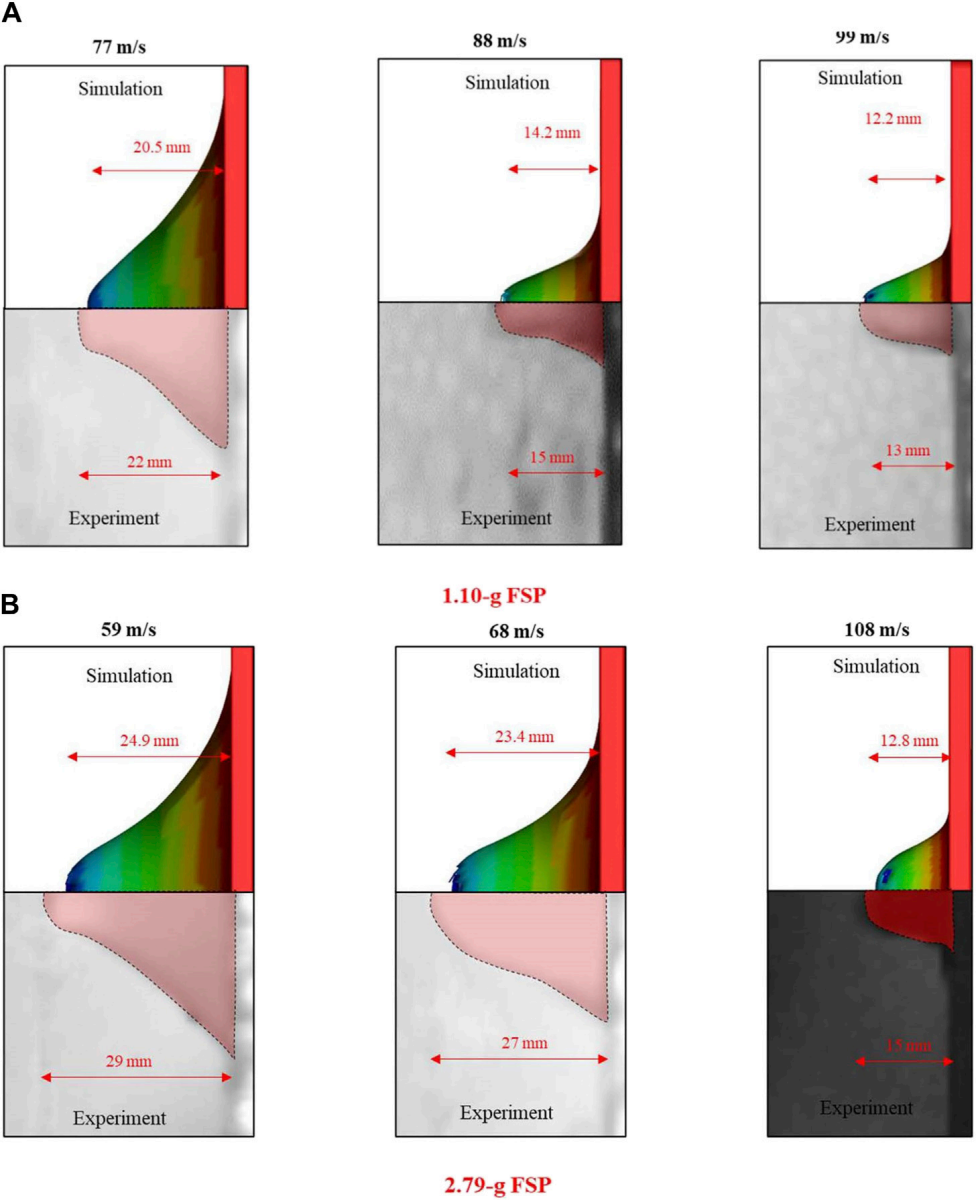
Peak displacements of the skin simulant in the direction of impact for different impact velocities (V_i_): **(A)** 1.10 g and **(B)** 2.79 g FSPs. The first, second, and third columns of each sub-part correspond to impact velocities below, similar to, and above the threshold velocity (V_th_), respectively. The direction of travel of the FSP is from right to left in the images. In the experiment images, the red area is the stretched skin simulant, black and dark gray areas are parts of the metal fixture, and light gray area is the background of the experimental setup.

Figure 6 shows the peak displacements (deformations) of the skin simulant in the direction of impact for various V_i_ values. Results corresponding to representative V_i_ values below (column i), similar to (column ii), and above (column iii) V_th_ are depicted. The simulation and experimental results are shown in the upper and lower halves of each panel, respectively. A reasonable qualitative and quantitative agreement was obtained between the experiment and simulation for each case, with the differences in peak displacements between the experiments and simulations being <1.5 mm (i.e., <5%) for the 1.10 g FSP and <4 mm (i.e., <15%) for the 2.79 g FSP. Moreover, the peak displacements in the experiments and simulations occurred at reasonably similar time points. The peak displacement of the skin simulant was a function of V_i_. Interestingly, the peak displacement of the skin simulant during perforation (higher impact velocities, column iii) of the FSP was lower than that during non-perforation (lower impact velocities, column i).

#### 3.1.3 Failure mechanism

**FIGURE 7:**
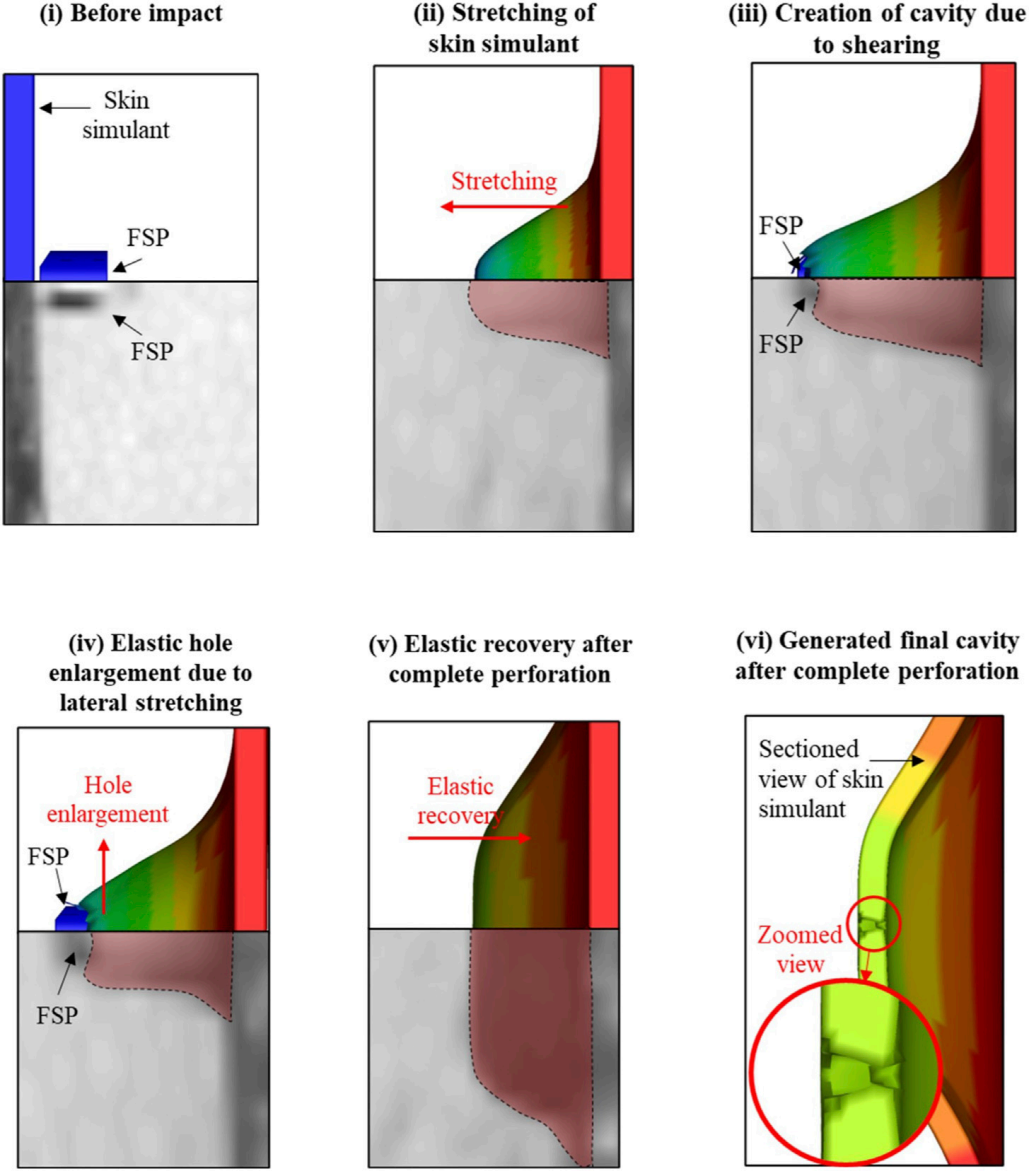
Failure process during the interaction of the FSP with the skin simulant.

Figure 7 shows the typical failure mechanism during the interaction of the FSP with the skin simulant. In each panel, the simulation image (upper half) is presented along with the corresponding experimental image (lower half). The skin simulant failed under the combination of shearing and elastic hole enlargement. Upon initial impact, the FSP stretched the skin simulant to a certain extent (Figure 7ii). Thereafter, the FSP sheared the skin simulant, resulting in the creation of a cavity (Figure 7iii); this was followed by lateral stretching of the skin simulant (Figure 7iv), a phenomenon typically known as elastic hole enlargement (Rosenberg et al., 2012). After complete perforation of the FSP, the laterally stretched skin simulant retracted elastically (Figure 7v). The combination of cavity creation in the stretched state followed by elastic retraction resulted in the final cavity size being smaller than the FSP size (Figure 7vi). The failure patterns between the experiments and simulations were similar.

**FIGURE 8:**
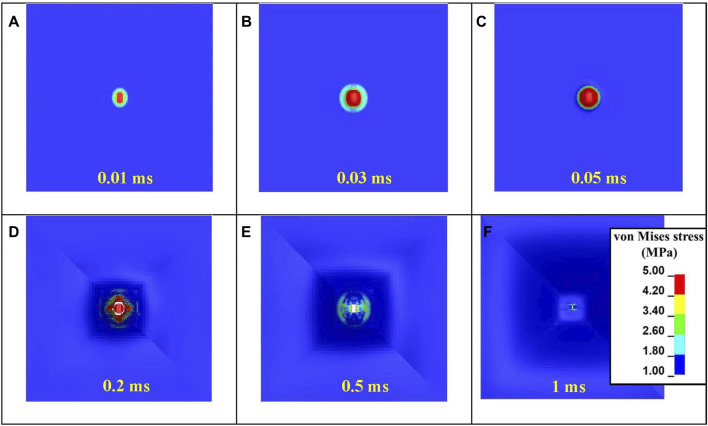
Evolution of the von Mises stress during various stages of the interaction of the FSP with the skin simulant: **(A)** initial contact; **(B, C)** stretching of the skin simulant; **(D)** cavity generation; **(E)** elastic recovery after complete perforation; **(F)** final cavity generated after complete unloading.

The mechanism of skin simulant failure is further illustrated using the von Mises stress distribution on the skin simulant’s rear surface (Figure 8). The stress concentration was pronounced in the vicinity of the impact zone. As the FSP made initial contact with the skin simulant, its rectangular nose engaged with the skin simulant, generating an elliptical stress contour (Figure 8A). Subsequently, as the skin simulant continued to stretch, the circular section of the FSP came into contact with the simulant, resulting in a circular stress contour (Figures 8B,C). The elements of the skin simulant beneath the impacting face of the FSP experienced extensive stretching, causing them to reach the failure strain. This marked the initiation and propagation of failure (Figure 8D). Once the element failed in the direction of the thickness, the skin simulant started unloading, resulting in elastic recovery (Figure 8E). The localized failure of the skin simulant (Figure 8D) followed by elastic recovery (Figure 8E) produced the final cavity, whose size was smaller than that of the FSP (Figure 8F).

### 3.2 Parametric studies

#### 3.2.1 Sensitivity of the skin simulant response to the input stress–strain curve

We investigated the sensitivity of the skin simulant response (i.e., V_th_ and peak displacement) to the input stress–strain curve. By default, the stress–stain curves corresponding to four strain rates (i.e., quasi-static, 1,000 s^-1^, 2,800 s^-1^, and 4,200 s^-1^) were used as inputs. In subsequent simulations, the stress–strain curve corresponding to a single strain rate was used as the input (i.e., 1,000 s^-1^, 2,800 s^-1^, or 4,200 s^-1^).

**FIGURE 9:**
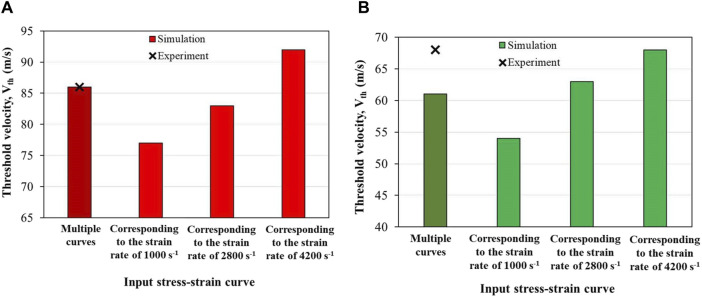
Sensitivity of the threshold velocity (V_th_) to the input stress–strain curve with **(A)** 1.10 g and **(B)** 2.79 g FSPs.

Figure 9 shows the V_th_ values corresponding to the aforementioned cases, which were sensitive to the input stress–strain curves and were hence rate dependent. The maximum value of V_th_ was obtained when the stress–strain curve corresponding to the highest strain rate was used. The V_th_ values from the simulations best matched with the experimentally obtained V_th_ when multiple stress–strain curves corresponding to the full spectrum of strain rates (i.e., quasi-static, 1,000 s^-1^, 2,800 s^-1^, and 4,200 s^-1^) were used.

**FIGURE 10:**
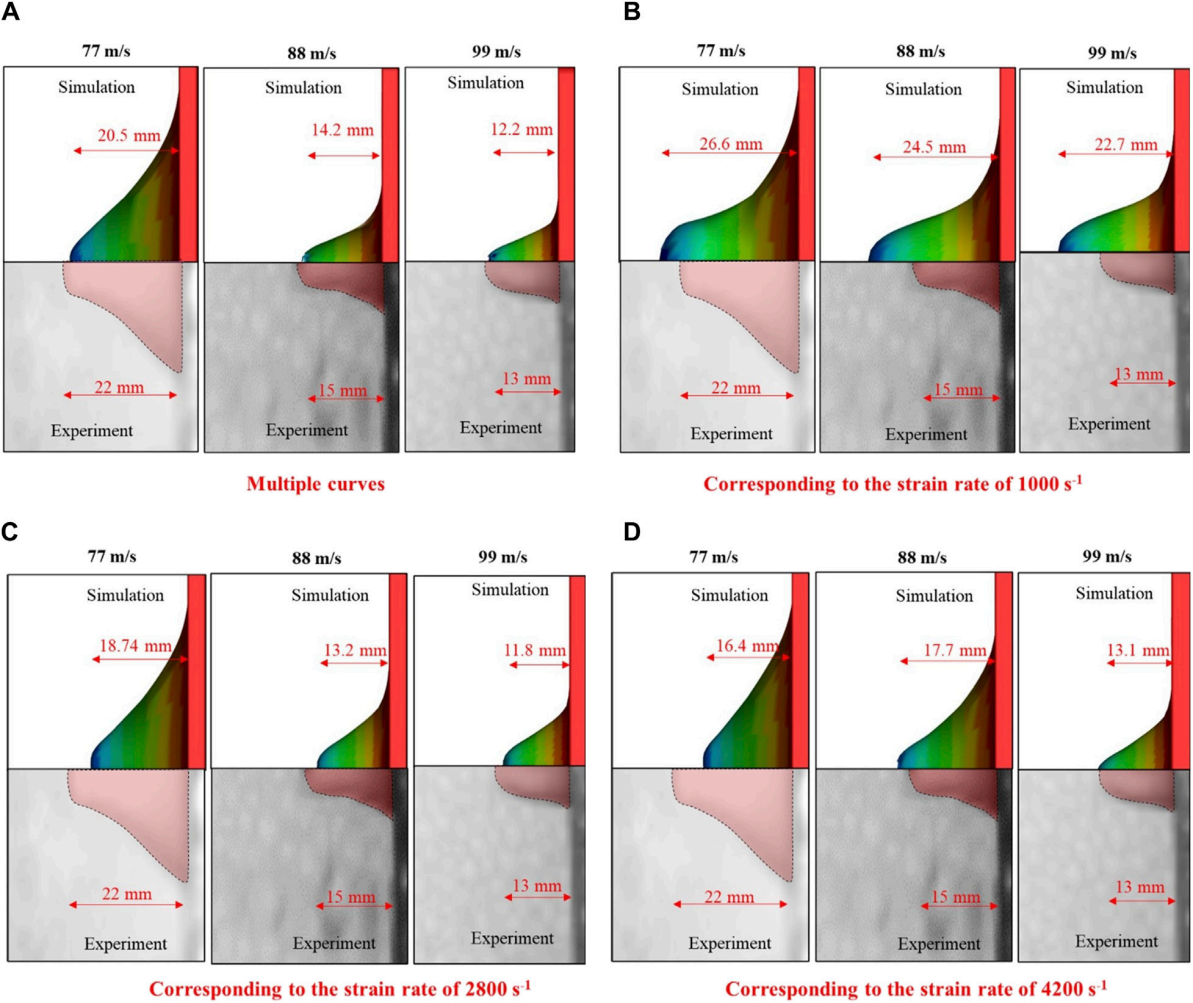
Peak displacement of the skin simulant with respect to input stress-strain curve **(A)** multiple curves **(B)** corresponding to the strain rate of 1000 s^−1^ **(C)** corresponding to the strain rate of 2800 s^−1^ **(D)** corresponding to the strain rate of 4200 s^−1^. Representative results for 1.10-g FSP are depicted. Similar trends are observed for 2.79-g FSP.

Strain-rate-dependent behavior was also observed in the peak displacement of the skin simulant. The peak displacements (Figure 10) of the skin simulant at various V_i_ values were in reasonable agreement with the experimental findings when multiple stress–strain curves corresponding to the full spectrum of strain rates were used. When a single stress–strain curve corresponding to a strain rate of 1,000 s^-1^ was used, the peak displacements corresponding to velocities of 88 m s^–1^ and 99 m s^–1^ were overpredicted. On the contrary, when a single stress–strain curve corresponding to the strain rate of either 2,800 s^-1^ or 4,200 s^-1^ was used, the peak displacement corresponding to the velocity of 77 m s^–1^ was underpredicted.

#### 3.2.2 Sensitivity of the skin simulant response to the input failure strain

**FIGURE 11:**
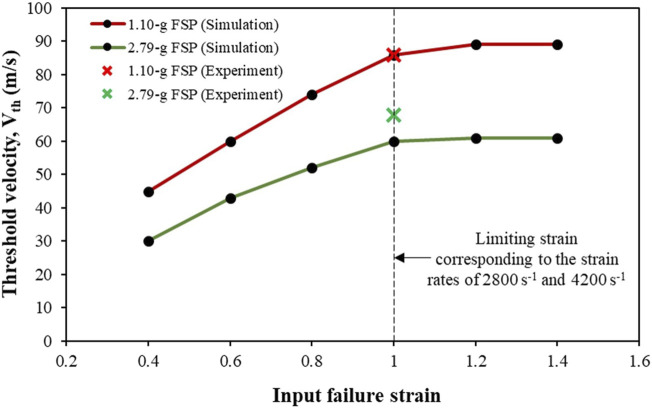
Sensitivity of the threshold velocity (V_th_) to the input failure strain values.

Figure 11 shows the sensitivity of V_th_ to the input failure strain. Note that the developed strain rates in the simulations at the investigated V_i_ values were in the range of 2,500–4,500 s^-1^. The limiting strain corresponding to input strain rates of 2,800 s^-1^ and 4,200 s^-1^ was ∼1 mm mm^–1^. Hence, the failure strain of ∼1 mm mm^–1^ at these strain rates varied for the parametric studies. When the input failure strain was based on the limiting strain estimated by the Gent model, the difference in V_th_ between the simulation and the experiment was not significant (<10%). However, when the failure strain was less than the limiting strain, the difference in V_th_ between the simulation and experiment was pronounced. For example, for input failure strains of 0.8, 0.6, and 0.4, the V_th_ from the simulations were underpredicted by ∼20%, ∼33%, and ∼52%, respectively, as compared to the V_th_ obtained experimentally. A near-plateau trend in the V_th_ was observed when the input failure strain exceeded the limiting strain.

#### 3.2.3 Effects of FSP velocity (constant mass) and FSP mass (constant impact energy)

**FIGURE 12:**
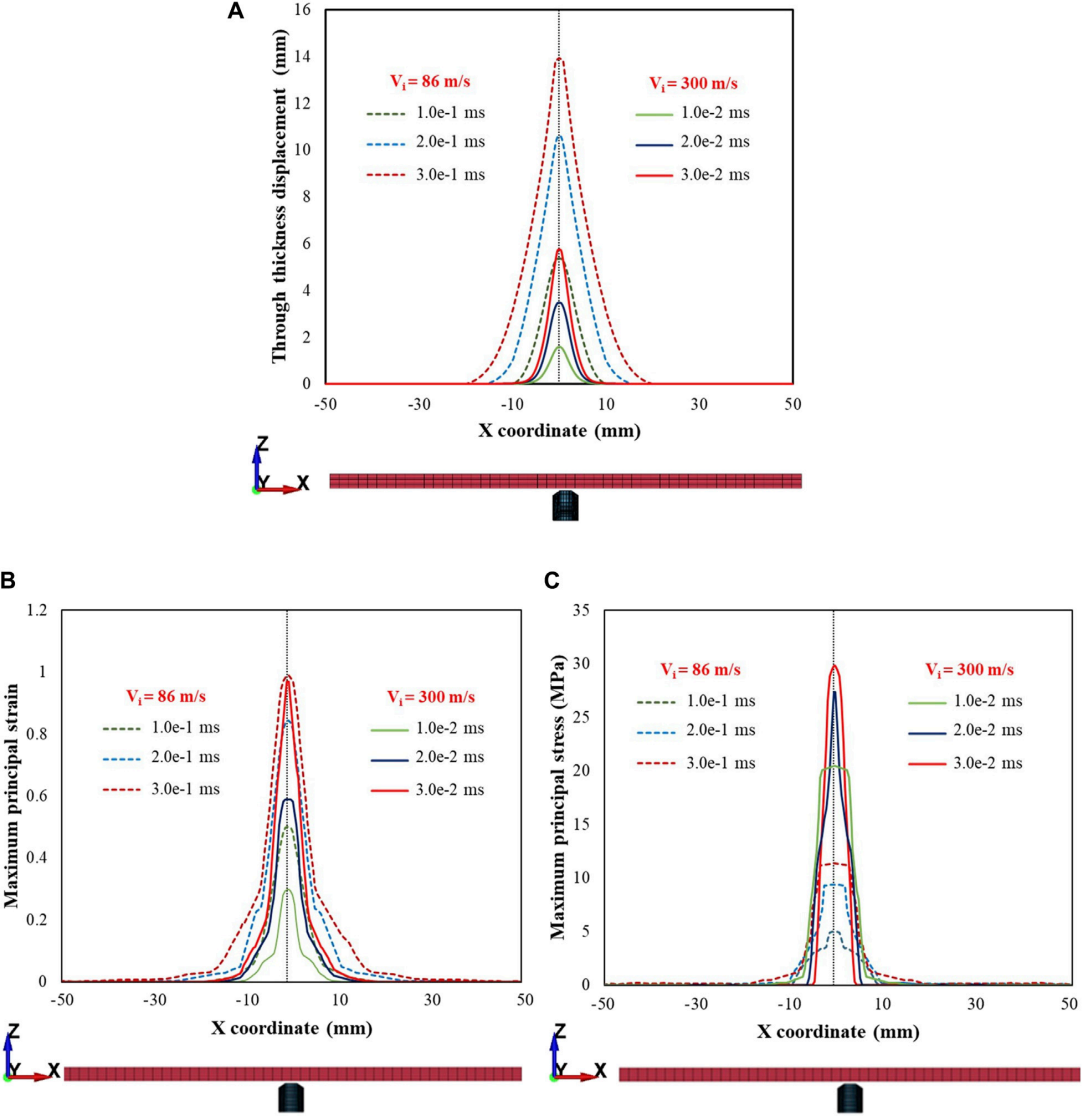
Plots depicting **(A)** displacement in the direction of impact (i.e., through thickness displacement), **(B)** maximum principal strain, **(C)** maximum principal stress within a cross section of the rear face for impact velocities (V_i_) of 86 and 300 m s^–1^. Note that z represents the thickness direction and x, y are the in-plane directions representing the cross section. Plots are depicted for the various time points during penetration. Note that there is a time scale separation between the impact velocities at 86 and 300 m s^–1^. Representative results along the x-axis for the 1.10 g FSP are presented. Similar trends were observed along the y-axis and for 2.79 g FSP.

Figure 12 shows the displacements of the skin simulant in the direction of impact (Figure 12A), maximum principal strain (Figure 12B), and maximum principal stress (Figure 12C) within a cross section (c/s) on the rear face. Representative results along the x-axis are shown for the 1.10 g FSP. Results for V_i_ = 86 m s^–1^ (low value corresponding to the V_th_ of the 1.10 g FSP) and V_i_ = 300 m s^–1^ (relatively high value) are presented, showing notable distinctions between the low and high V_i_ cases. The FSP with high V_i_ induced relatively smaller amplitude deformations (solid lines in Figure 12A) and higher stresses (solid lines in Figure 12C) over a localized area, facilitating relatively easier penetration of the skin simulant over a shorter time (∼0.03 ms). For the low V_i_ case, relatively larger amplitude deformations (dotted lines in Figure 12A) and lower stresses (dotted lines in Figure 12C) were developed. Interestingly, the penetration process for low velocity required approximately one order of magnitude more time (∼0.3 ms) than the high-velocity impact. Hence, the deformation and stress encompassed a relatively larger c/s area. Similar trends were observed along the y-axis and for the 2.79 g FSP.

**FIGURE 13:**
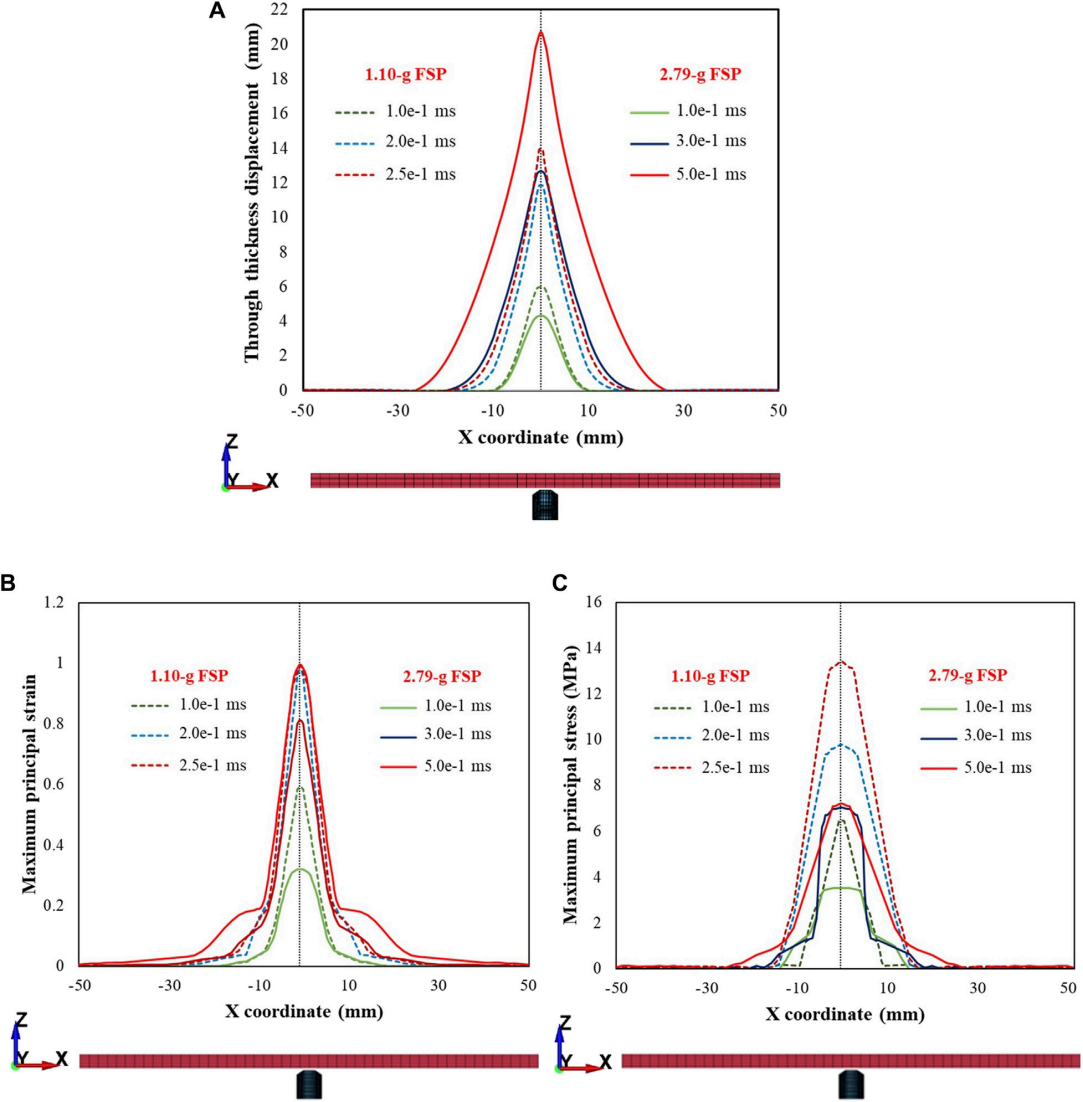
Plots depicting **(A)** displacement in the direction of impact (i.e., through thickness displacement), **(B)** maximum principal strain, **(C)** maximum principal stress within a cross section of the rear face for the same impact energy level (5 J) for the 1.10 g and 2.79 g FSPs. Note that z represents the thickness direction and x,y are the in-plane directions representing the cross section. Plots are depicted for the various time points during penetration.

When the mass of the FSP was changed (Figure 13) while maintaining the same impact energy (i.e., 5 J), the FSP with a smaller mass induced relatively smaller amplitude deformations (dotted lines in Figure 13A) and higher stresses (dotted lines in Figure 13C) over a localized area. Thus, for the same impact energy, the FSP with the smaller mass achieved relatively easier perforation due to the higher V_i_.

#### 3.2.4 Effect of the FSP material

Table 5 shows the V_th_ and E_th_/A values when the FSP material was changed from mild steel to BB and PVC plastics. The mass of the FSP was maintained constant (i.e., 1.1 g). Owing to the differences in the densities of BB and PVC plastics with respect to mild steel, the sizes of the BB and PVC FSPs increased proportionally even as the same shape of the FSP was maintained. Compared to the V_th_ of the mild steel FSP, the V_th_ values of BB and PVC FSPs were higher by ∼22% and ∼33%, respectively. These increases in V_th_ are attributed to the larger cross-sectional areas (Table 5), which result in load distributions over wider areas.

**TABLE 5:** Effects of the FSP materials on the threshold velocities (V_th_). Results are presented for the 1.10 g FSPs.

FSP material	Cross-sectional area, A (mm^2^)	V_th_ (m/s)	E_th_/A (J mm^2^)
Mild steel	22.72	86	0.18
BB plastic	56.00	105	0.11
PVC plastic	73.60	115	0.10

**FIGURE 14:**
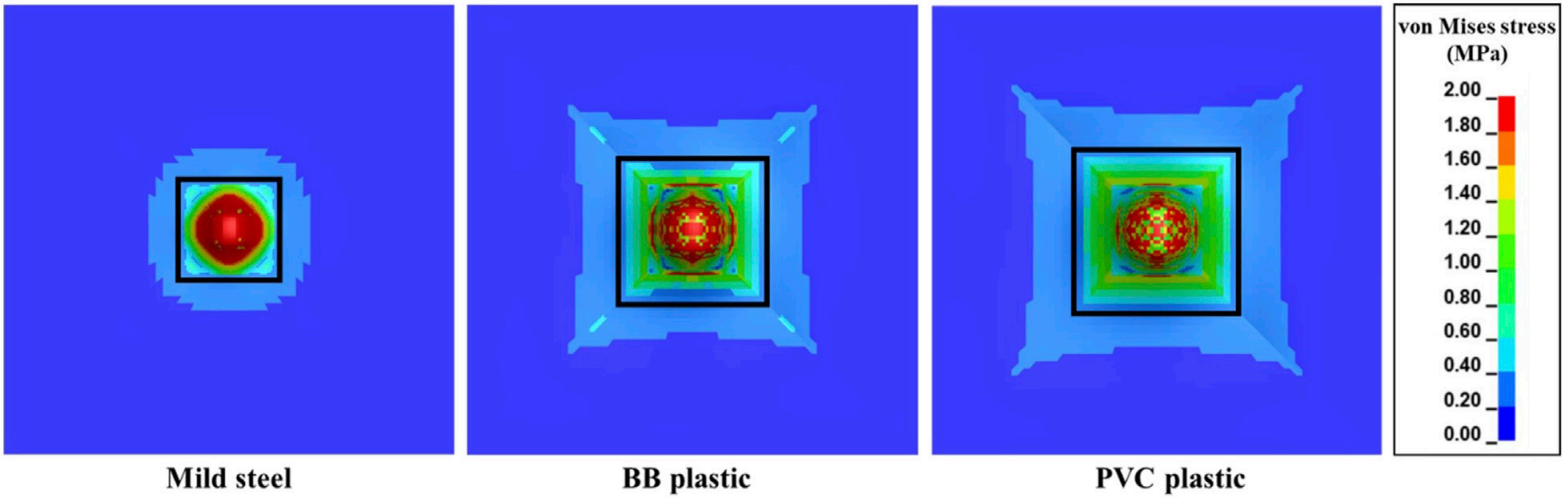
von Mises stress contours of the skin simulant during interaction with FSPs made of different materials for an impact velocity (V_i_) of 86 m s^–1^. The von Mises stress in the region bounded by the black rectangle is above the laceration threshold of 1 MPa, indicating increases in the affected area for FSPs made of BB and PVC plastics.

Compared to the E_th_/A of the mild steel FSP, the E_th_/A of BB and PVC FSPs were lower by ∼39% and ∼44%, respectively. Despite the reductions in E_th_/A with respect to mild steel, the E_th_/A values of BB (0.11 J mm^–2^) and PVC (0.10 J mm^–2^) exceeded the proposed contusion threshold of 0.0252 J mm^–2^ (Park et al., 2011). Furthermore, considerable stresses were generated (e.g., above the laceration threshold of 1 MPa (Park et al., 2011)) over a larger area (Figure 14).

#### 3.2.5 Effect of the skin simulant thickness

**FIGURE 15:**
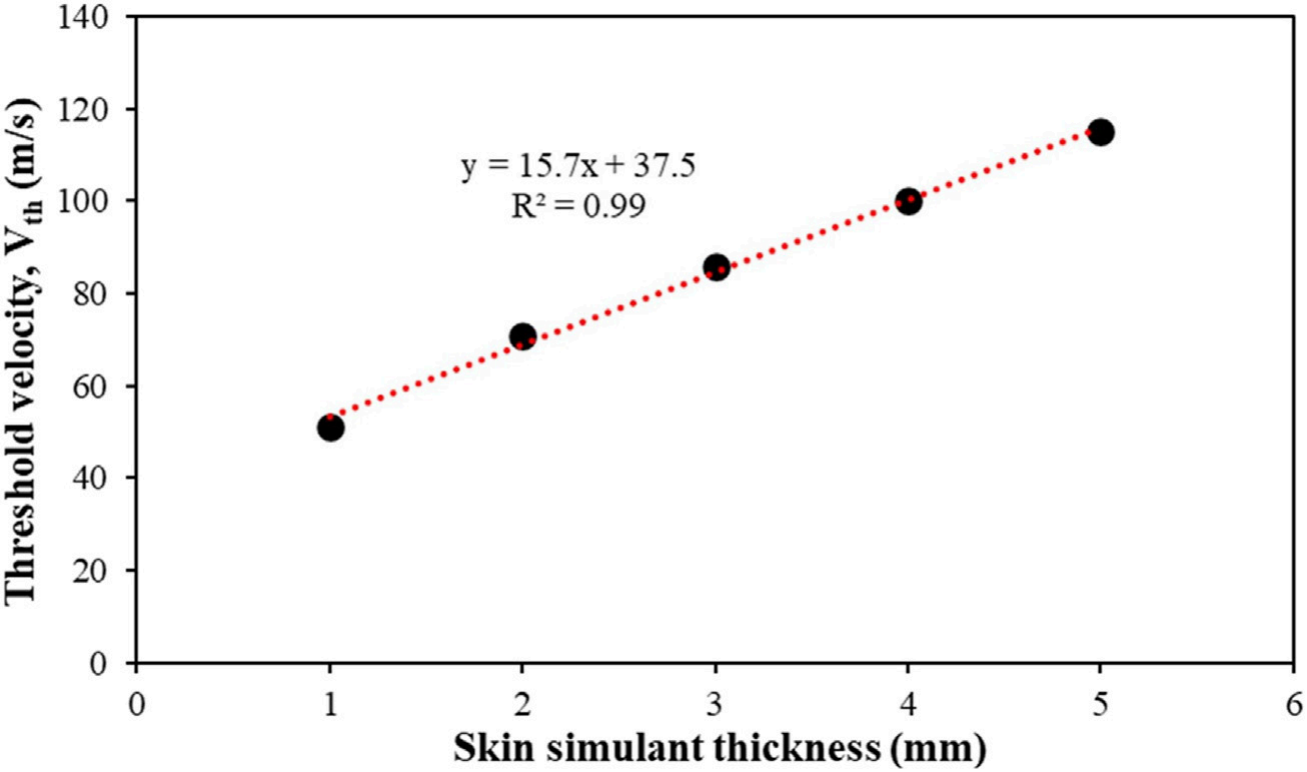
Effect of skin simulant thickness on the threshold velocity (V_th_). Results are presented for the 1.10 g FSP.

Figure 15 shows V_th_ as a function of the skin simulant thickness, which exhibits a linear relationship (R^2^ = 0.99).

## 4 Discussion

In this work, the responses of the skin simulant to ballistic impact were investigated using experiments and concurrent simulations. Simulations were further used to conduct parametric studies by incorporating the rate-dependent material responses at different strain rates (i.e., quasi-static, 1,000 s^-1^, 2,800 s^-1^, and 4,200 s^-1^). Note that the developed strain rates in the simulations at the investigated impact velocities were in the range of 2,500–4,500 s^-1^. Hence, the stress–strain curves at the chosen strain rates (i.e., 1,000–4,200 s^-1^) were found to be suitable and related to the experimental velocity ranges. The full spectrum of stress–strain responses at the aforementioned strain rates were used as the inputs unless stated otherwise.

The V_th_ and E_th_/A values decreased as the FSP size increased (Table 4). This trend is consistent with observations in literature. For instance, Breeze et al. (2013) estimated V_50_ values (i.e., velocity corresponding to 50% probability of perforation) of chisel-nosed FSPs weighing 0.16 g, 0.49 g, and 1.10 g for perforation of goat skin and noted that the 0.16 g and 1.10 g FSPs offered the highest and lowest V_50_ values, respectively. Breeze and Clasper (2013) also compiled data from various experiments involving fragment impact on either skin or skin simulant, and their comparative analysis revealed a consistent inverse relationship between the fragment size and V_50_ value. Furthermore, the range of E_th_/A (i.e., 0.12–0.18 J mm^–2^) obtained in this work is commensurate with that reported in literature for skin or skin simulant perforation (Kneubuehl, 2011; Bir et al., 2012; Jin et al., 2019).

We observed that the V_th_, E_th_/A, and peak displacement values of the skin simulant were sensitive to the strain rate (Figures 9, 10). These results demonstrate an interesting paradigm based on the input stress–strain curve. The aforementioned values from the simulations matched reasonably well with those from the experiments (Figures 5, 6; Table 4) when a full spectrum of stress–strain responses at different strain rates were used as the inputs. This is because the material model appropriately interpolates the data at the strain rates realized in the simulations based on the input stress–strain curves. When a single curve was applied, the results did not match with those from experiments. The V_th_ was underpredicted and overpredicted when stress–strain curves corresponding to strain rates of 1,000 s^-1^ and 4,200 s^-1^ were used as inputs, respectively (Figure 9). When a single stress–strain curve corresponding to a strain rate of 1,000 s^-1^ was used, the peak displacements at higher impact velocities (i.e., 88 and 99 m s^–1^) were overpredicted. On the contrary, when a single stress–strain curve corresponding to a strain rate of either 2,800 s^-1^ or 4,200 s^-1^ was used, the peak displacement at a lower V_i_ (i.e., 77 m s^–1^) was underpredicted (Figure 10). These responses are attributed to stiffening of the material with increase in the strain rate. Upadhyay et al. (2020, 2021) also reported a similar rate-dependent stiffening response in a similar silicone-based soft material. Similar rate-dependent behaviors were also observed in polymers (Li and Lambros, 2001; Chen et al., 2024). Our results underscore that the strain-rate-dependent material response should be incorporated when modeling skin and skin surrogates under ballistic impact. Currently, very few models incorporate strain-rate-dependent behaviors (see Joodaki and Panzer (2018) and the references therein; Liu et al., 2014).

The failure mechanism of the skin simulant involved shearing followed by elastic hole enlargement (Figures 7, 8). The skin simulant stretched in the direction of impact of the FSP until it reached the failure strain (Figure 7i–iii). The subsequent unloading phase involved lateral stretching by the FSP as the skin simulant attempted to undergo elastic recovery but was constrained by the presence of the FSP (Figure 7iv), a phenomenon known as elastic hole enlargement. After complete perforation (Figure 7v), the final size of the generated cavity induced by shearing remained smaller than the diameter of the FSP (Figure 7vi). This occurrence of a smaller cavity in the skin simulant compared to the FSP size due to elastic retraction is consistent with findings documented in existing literature (Inchingolo et al., 2011; Kneubuehl et al., 2011; Baptista et al., 2014; Carr et al., 2014; Serraino et al., 2020).

Interestingly, we observed that the peak displacements of the skin simulant at impact velocities corresponding to perforation were lower than those corresponding to non-perforation (Figures 6, 10). As the velocity of the FSP increases, the rate of loading increases and failure strain decreases (Li and Lambros, 2001; Shergold et al., 2006; Lim et al., 2011; Khatam et al., 2014; Ottenio et al., 2015; Joodaki and Panzer, 2018; Chen et al., 2024). Hence, the deformation becomes more localized with a relatively smaller amplitude (Figures 12A, 13A) and generates high stresses (Figures 12C, 13C) over a small area in a short time. This suggests that failure is stress driven (for additional details, see Supplementary Material), facilitating relatively easier perforation of the skin simulant at velocities corresponding to perforation (Figures 12, 13). The shorter times required for perforation at higher velocities result in relatively smaller peak displacements of the skin simulant compared to those at lower velocities (Figures 6, 10).

The V_th_ values were sensitive to the input failure strain (Figure 11). Owing to the absence of experimental data on failure strain, we estimated the failure strains using the Gent model, which gives the limiting or maximum extensibility. The V_th_ from the simulation matched the experimental V_th_ (Figure 11) when the input failure strain was based on the estimate from the Gent model (or within 10% of the estimates from the Gent model). For input failure strain values lower than those estimated by the Gent model, the V_th_ values were underpredicted. The V_th_ exhibited a near-plateau trend when the input failure strain was above the limiting strain estimated by the Gent model. This underscores the importance of the input failure strain value. Our results suggest that the limiting strain obtained from the Gent model is a reasonable estimate of the failure strain. This is especially noteworthy considering the typical lack of high-strain-rate experimental data up to failure strain in literature (Joodaki and Panzer, 2018).

We investigated the influence of the FSP material on the ballistic responses of the skin simulant (Table 5). FSPs made of three different materials (mild steel, BB plastic, and PVC plastic) and having the same mass (1.10 g) were studied. Even though V_th_ increased by 22%–33% with the plastic FSPs, these increases may not be sufficient to qualify plastic FSPs as non-lethal. A few investigations suggest that projectiles made of BB plastic can penetrate the skin (Grocock et al., 2006; Tsui et al., 2010; Jin et al., 2019) when expelled with considerable velocity (90–160 m s^–1^). Furthermore, the energy densities (Table 5) of the plastic FSPs exceeded the contusion threshold of 0.0252 J mm^–2^ (Park et al., 2011) and generated stresses in the skin simulant by exceeding the laceration threshold of 1 MPa (Park et al., 2011) (Figure 14). Considerable stresses were also generated over larger areas. These observations are critical as plastic projectiles are generally used as non-lethal projectiles to control or disperse crowds during law enforcement (Bir et al., 2005; Rezende-Neto et al., 2009; Bir et al., 2012).

We also found a linear relation between V_th_ and skin simulant thickness (Figure 15). In this work, we used narrow ranges of velocities (60–100 m s^–1^) and masses (1.10 g; 2.79 g) of chisel-nosed FSPs. Hence, in this work, the linear relationship between V_th_ and skin simulant thickness is independent of the FSP velocity and mass. In the future, it would be interesting to investigate whether the linear relation between V_th_ and skin simulant thickness is applicable for wider ranges of velocities, masses, and shapes of the FSPs.

## 5 Limitations

The present work has a few limitations. In this work, similar stress–strain responses were assumed under compression and tension; this is mainly due to the lack of compression data on the skin simulant used in this work. It should be noted that soft polymeric materials of this class often behave asymmetrically under tension and compression (Donato and Bianchi, 2012; Pellegrino et al., 2015; Siviour and Jordan, 2016; Chen et al., 2024). Efforts will therefore be made in the future to obtain and utilize compression stress–strain data for the skin simulant.

## 6 Conclusion

In this study, we investigated the rate-dependent ballistic responses of the skin simulant under fragment impact. A finite-element model was developed alongside experimental testing, and reasonable agreement was observed between the numerical simulation and experimental results. The following key conclusions are drawn from this study.

• The threshold velocity (V_th_) and energy density (E_th_/A) decrease with increasing size of the FSP.
• The energy density (E_th_/A) was in a narrow range (0.12–0.18 J mm^–2^) for investigated FSPs.
• The V_th_ and peak displacement of the skin simulant exhibited sensitivity to the strain rate. V_th_ was underpredicted when using a single stress–strain curve corresponding to a strain rate of 1,000 s^-1^ and overpredicted when using a single stress–strain curve at a strain rate of 4,200 s^-1^. The closest match between the simulated and experimental V_th_ was achieved when the stress–strain curves were considered across the full spectrum of strain rates (quasi-static, 1,000 s^-1^, 2,800 s^-1^, and 4,200 s^-1^). Similar trends were observed for the peak displacement of the skin simulant.
• The peak displacement of the skin simulant was a function of the impact velocity. The peak displacements of the skin simulant at lower impact velocities (during non-perforation) were higher compared to those at higher velocities (during perforation). This was attributable to the stress-driven failure.
• The failure mechanism of the skin simulant primarily entailed cavity shearing followed by elastic hole enlargement. The final size of the resulting cavity remained smaller than the size of the corresponding FSP.
• The V_th_ from simulation best matched the experimental V_th_ when the input failure strain was close to the limiting strain estimated from the Gent model.
• Although 1.10 g FSPs made of BB and PVC plastics demonstrated higher V_th_ than mild steel FSPs of the same size, they exhibited significant threats of contusion and laceration.
• A linear relationship was noted between V_th_ and skin simulant thickness.

## Data Availability

The original contributions presented in the study are included in the article/Supplementary Material, and any further inquiries may be directed to the corresponding author.

## Abbreviations

FSP, fragment simulating projectile; V_i_, impact velocity; V_r_, residual velocity; V_th_, threshold velocity; E_th_, threshold energy; A, cross-sectional (c/s) area; E_th_/A, energy density; BB, ball bearing; PVC, polyvinyl chloride; SHPB, split Hopkinson pressure bar.
