# Supplementary material for: Investigation of dynamic responses of skin simulant against fragment impact through experiments and concurrent computational modeling
Source: Front Bioeng Biotechnol. 2024 Aug 27;12:1422685. doi: 10.3389/fbioe.2024.1422685 (PMC11383784; doi:10.3389/fbioe.2024.1422685)
Supplement: Supplementary file 1 [file DataSheet1.docx]

**Supplementary material**

**Mesh convergence:**

Fig. S1 shows the variation in residual velocity (V_r_) of 1.10-g FSP as a function of element size at the representative impact velocity (V_i_) of 160 m/s.


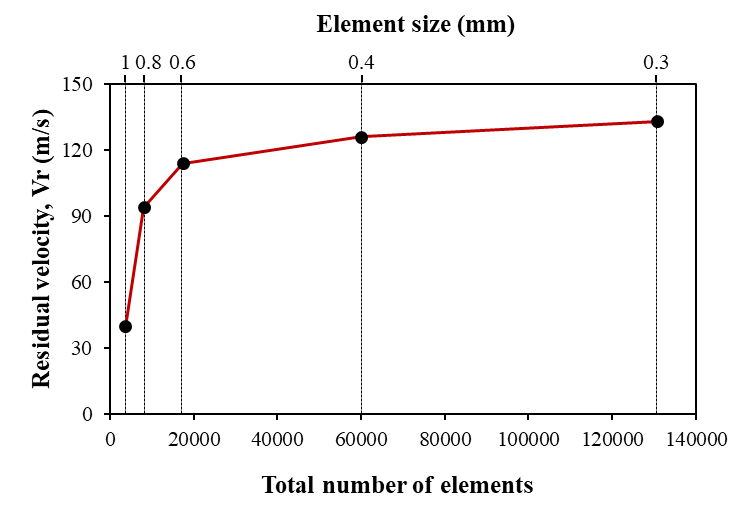


**Fig. S1.** Variation in residual velocity (V_r_) as a function of element size

**Protocol used while shading experimental images:**

The shaded red areas in the figures showing experiment images are marked through manual delineation. The stretched part of the skin simulant in experiments was highlighted with these red regions for better visualization due to the blurring of high-speed images after zooming in. This process involved the following steps.

Step 1. Zooming and cropping of high-speed images to show the stretched skin simulant. The stretched skin simulant is pointed through red arrows (Fig. S2 (i)).

Step 2. Manually tracing the boundary of stretched skin simulant. The traced boundary is visible as dotted black lines (Fig. S2 (ii)).

Step 3. Shading the area under the traced boundary of the stretched skin simulant to enhance the visualization. A shaded area of stretched skin simulant in red color can be seen in Fig. S2 (iii).


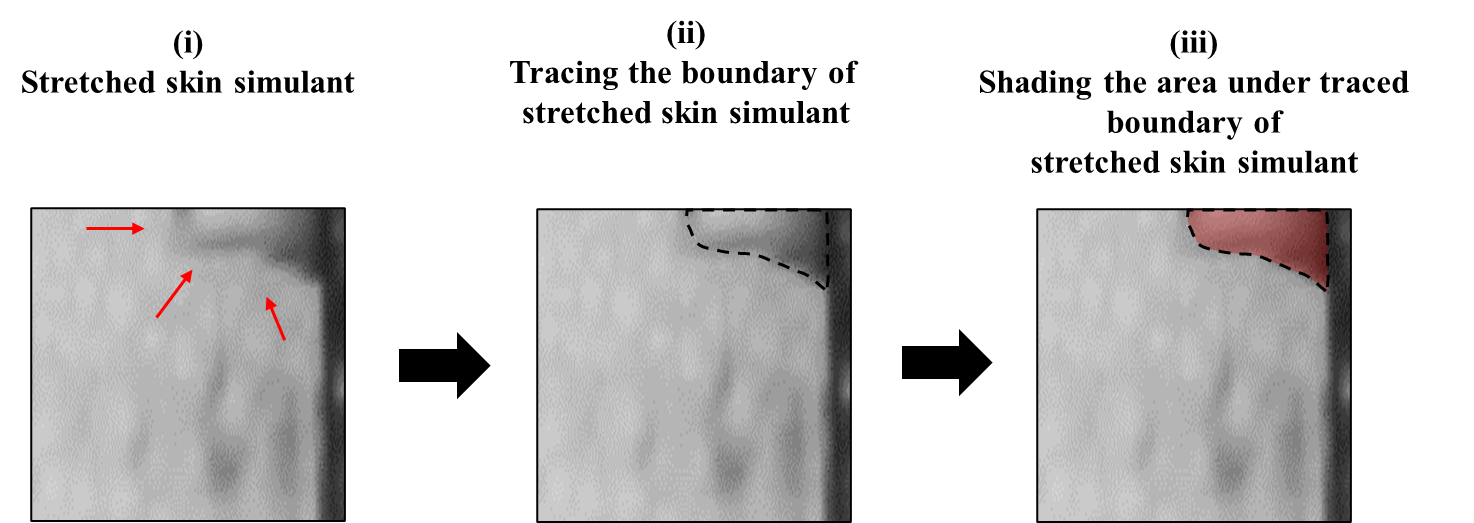


**Fig. S2.** Images in a sequence depicting the protocol followed for shading experiment images

**Stress driven failure:**

Our results indicate that the failure is strain rate dependent, and stress rather than strain dictates the failure during perforation. As the strain rate increases, the failure stress increases, and strain to failure decreases (Table 1, Joodaki and Panzer 2018; Khatam et al. 2014; Lim et al. 2011; Ottenio et al. 2015; Shergold et al. 2006). This means that the material strained at a higher strain rate will fail at smaller deformation values (Fig 12 (a), Fig. 13 (a)) than the same material strained at a lower strain rate. However, for the same material, the failure stress corresponding to the higher strain rate will be larger than the failure stress (Fig 12 (c), Fig. 13 (c)) corresponding to the lower strain rate. Such a failure at high-strain rates is typically called a “stress driven failure”.

**References:**

Joodaki H, Panzer MB (2018) Skin mechanical properties and modeling: A review Proceedings of the Institution of Mechanical Engineers, Part H: Journal of Engineering in Medicine 232:323-343

Khatam H, Liu Q, Ravi-Chandar K (2014) Dynamic tensile characterization of pig skin Acta Mechanica Sinica 30:125-132

Lim J, Hong J, Chen WW, Weerasooriya T (2011) Mechanical response of pig skin under dynamic tensile loading International Journal of Impact Engineering 38:130-135

Ottenio M, Tran D, Annaidh AN, Gilchrist MD, Bruyère K (2015) Strain rate and anisotropy effects on the tensile failure characteristics of human skin Journal of the mechanical behavior of biomedical materials 41:241-250

Shergold OA, Fleck NA, Radford D (2006) The uniaxial stress versus strain response of pig skin and silicone rubber at low and high strain rates International journal of impact engineering 32:1384-1402
